# Attention Networks in Deaf Youth: A Cross-Sectional Study of Age-Related Patterns

**DOI:** 10.3390/jintelligence14100242

**Published:** 2026-10-03

**Authors:** Tongao Zeng, Fuxiang Li, Haifeng Lyu, Ziyao Liu, Hongli Xiao, Bin Du, Zhaoying He, Fangfang Liu, Han Zhu

**Affiliations:** 1 Department of Special Education, Faculty of Education, East China Normal University, Shanghai 200062, China; 52274107002@stu.ecnu.edu.cn (T.Z.); 51264107013@stu.ecnu.edu.cn (F.L.); freizliu@stu.ecnu.edu.cn (Z.L.); 52264107004@stu.ecnu.edu.cn (H.X.); 2 School of Lifelong Education, Nanjing University, Nanjing 210093, China; cxspecedu@yeah.net; 3 Department of Psychology, Faculty of Education, China West Normal University, Nanchong 637009, China; dubin@cwnu.edu.cn; 4 Graduate School of Education, Peking University, Beijing 100871, China; zyhe2025@pku.edu.cn

**Keywords:** attention networks, executive control, EZ-diffusion modeling, deafness, childhood, adolescence

## Abstract

Attention plays a central role in intellectual development and social interaction. Attention may differ between deaf and hearing youth, but it remains unclear whether such differences reflect hearing status, language experience, age-related factors, or some combination of these factors. In this cross-sectional study, we combined the Attention Network Test with comparisons of alternative age functions, age-stratified analyses, and EZ-diffusion modeling using data from 525 participants aged 6–19 years (200 deaf and 325 hearing). Both groups performed similarly during childhood, and alerting showed no reliable cross-sectional association with age, with no reliable difference between groups. In both groups, executive-control costs were smaller at older ages. The Bayesian information criterion (BIC) favored a linear age function for deaf participants and a logarithmic function for hearing participants. Crucially, however, the formal Group × Age interaction was not significant; the difference in functional form should therefore be treated as descriptive. By contrast, orienting was linearly related to age among deaf participants but followed a decelerating quadratic pattern among hearing participants; the Group × Age interaction was significant. Deaf participants also showed smaller benefits from spatial cues during early and middle adolescence. EZ-diffusion modeling further indicated that conflict produced a larger reduction in drift rate and a larger increase in non-decision time among deaf than among hearing participants, whereas conflict-related boundary adjustments were comparable between groups. Together, these findings suggest that deaf and hearing youth differ in visual attention in a network-specific, age-related manner rather than exhibiting either a generalized deficit or a generalized compensatory advantage. Because hearing status, language experience, and educational context were intertwined in this sample, the observed group differences cannot be interpreted as causal effects of deafness or auditory deprivation.

## 1. Introduction

Attention is a core cognitive mechanism that supports intellectual development, academic learning, and socioemotional development in children and adolescents (Cai et al., 2024; Lewis et al., 2018). Posner and Petersen proposed that attention is not a unitary construct but comprises three distinct networks—alerting, orienting, and executive control—that differ in their neural bases and cognitive functions (Fan et al., 2002; Fan et al., 2005; Wang & Fan, 2007). The alerting network establishes and maintains readiness for incoming information (Fernandez-Duque & Posner, 1997). The orienting network selects and prioritizes information at particular spatial locations or with particular sensory features (Posner, 1980). The executive control network handles conflict monitoring, error processing, and behavioral regulation when competing responses arise (Fan et al., 2002; Fan et al., 2009; Wang & Fan, 2007). These three networks work together during complex cognition but show distinct age-related patterns (Rueda & Posner, 2013).

Elements of all three attention networks are evident by the end of the first year of life, but their subsequent age-related courses differ across childhood and adolescence (Rueda & Posner, 2013). This asynchrony has been linked to gradual structural and functional maturation in cortical systems, particularly prefrontal and parietal regions (Barnea-Goraly et al., 2005; Konrad et al., 2005; Lenroot & Giedd, 2006; Schmithorst et al., 2002). Executive control has the most protracted course (Abundis-Gutiérrez et al., 2014; Huizinga et al., 2006). Inhibitory control and conflict processing improve rapidly in early childhood, especially around 6–7 years of age (Band et al., 2000; Casey et al., 2000; Mullane et al., 2016), and continue to improve through adolescence alongside synaptic pruning and network reorganization in the prefrontal cortex and anterior cingulate cortex (ACC). The rate of improvement then slows, with performance approaching stability in late adolescence or early adulthood (Boen et al., 2021). Orienting follows a different course: basic operations mature relatively early, whereas more demanding forms continue to change. ANT studies indicate that basic spatial orienting to exogenous cues and the associated cue benefits approach adult levels by about 6 years of age (Gupta & Kar, 2009; Mullane et al., 2016). Development extends further when endogenous control is required, particularly when attention must be disengaged from an invalidly cued location and redirected elsewhere (Abundis-Gutiérrez et al., 2014; Enns & Brodeur, 1989; Konrad et al., 2005). Higher-order disengagement and reorienting, supported mainly by the superior parietal lobule, temporoparietal junction, and frontal eye fields, may therefore continue to change through late childhood and adolescence (Rueda & Posner, 2013). Alerting also has a prolonged course. Basic responses are present in the first months of life, but the system continues to change during the school years (Lewis et al., 2018; Mullane et al., 2016; Pozuelos et al., 2014). Younger children often have difficulty sustaining tonic alertness without external support and consequently rely more on warning cues, producing larger phasic alerting effects in early childhood (Abundis-Gutiérrez et al., 2014; Pozuelos et al., 2014; Rueda et al., 2004). As the right frontoparietal network matures, the capacity for endogenous alerting strengthens and reliance on external cues declines. A gradual increase in frontal contingent negative variation (CNV) amplitude may reflect this process (Hämmerer et al., 2010; Padilla et al., 2014; Thillay et al., 2015). Cross-sectional and longitudinal studies have reported nonlinear age differences in alerting-related measures, including quadratic patterns (Giovannoli et al., 2021; Yan et al., 2018), although the functional interpretation of these patterns remains uncertain. The three networks therefore show nonidentical age-related courses in hearing samples. Whether deaf and hearing participants show different cross-sectional age functions remains unresolved.

For individuals with hearing loss, the absence of early auditory input can disrupt the formation of auditory cortical networks during critical developmental periods, thereby contributing to atypical central auditory processing and influencing visual attention (Campbell & Sharma, 2014; M. W. G. Dye & Bavelier, 2013; Lazard et al., 2013; Sharma & Mitchell, 2013; Shinn-Cunningham & Best, 2008). Accounts of visual attention in deaf populations differ in scope. The deficit hypothesis suggests broadly weaker performance across attention networks, whereas the compensation hypothesis proposes broadly enhanced visual attention following reduced auditory access. An integrative account instead suggests that group differences depend on the attention operation, task demands, age, and language or educational context (M. W. Dye & Bavelier, 2010; Daza & Phillips-Silver, 2013). The findings on visual attention networks in deaf populations are selective rather than uniform. Studies of deaf adults have reported enhanced covert exogenous orienting or phasic alerting in some tasks and larger flanker interference effects in others (Chen et al., 2010; M. W. Dye et al., 2007; Gioiosa Maurno et al., 2024; Holmer et al., 2020; Prasad et al., 2022). Findings in deaf children likewise vary by network, task, and age, ranging from differences in tonic alerting or basic orienting to heterogeneous executive-control results (Daza & Phillips-Silver, 2013; M. W. Dye & Hauser, 2014; M. W. Dye & Terhune-Cotter, 2023; Merchan et al., 2022; Pozuelos et al., 2014). Although these findings have advanced our understanding of attentional mechanisms in deaf individuals, differences in attention-network performance across age groups remain poorly understood. Importantly, most existing studies focus on a narrow age range spanning early to middle childhood, with relatively little attention to adolescence. Because adolescence is an important period of neurocognitive reorganization, this gap limits understanding of whether attention-network differences between deaf and hearing individuals vary across childhood and adolescence. The present study therefore evaluates whether group differences are network- and age-related.

More importantly, the diffusion decision model (DDM) conceptualizes binary perceptual choices as arising from momentary evidence that is sampled and accumulated over time (Ratcliff & McKoon, 2008). Full distributional diffusion models jointly model reaction-time distributions and response accuracy. In the present study, we used the EZ-diffusion approximation, which estimates drift rate (v), boundary separation (a), and non-decision time (Ter) from condition-level accuracy and the mean and variance of correct-trial RTs, rather than fitting the full RT distribution. Within this framework, observed choices can be decomposed into latent quantities, drift rate (v), boundary separation (a), and non-decision time (Ter), providing model-based summaries of performance that consider accuracy and RT together. These parameters provide a compact process-level summary of evidence accumulation, response caution, and non-decision processes while preserving the speed–accuracy trade-off within a formal decision model. Research applying diffusion models to attention paradigms commonly interprets drift rate as a model-based estimate of the quality or efficiency of evidence accumulation, whereas boundary separation is interpreted as a model-based estimate of response caution (White et al., 2011). In attention tasks, differences in drift rate may be associated with attentional selection or distractor processing, but drift rate is not a direct measure of either process. In deaf populations, Stoll and Dye (2019) reported differences in estimated drift rate across visual-field conditions without corresponding differences in boundary separation, illustrating how diffusion-model parameters can provide a complementary characterization of performance beyond mean RT and accuracy. However, two questions remain unresolved: whether deaf and hearing individuals differ in these parameters across this age range and whether previously reported group differences in attention-network scores reflect genuine processing inefficiency or strategic adjustments in the speed–accuracy trade-off. Accordingly, we applied the EZ-diffusion model (Wagenmakers et al., 2007) to compare estimated decision parameters across ANT conditions and groups, providing a complementary model-based description of attention-network performance. Combining these analyses allows us to characterize group differences in behavioral conflict costs and cue benefits, together with their cross-sectional age associations, while examining complementary estimates of decision parameters in the same sample. The EZ analyses distinguish overall group differences in these parameters from group differences in their modulation by conflict or cues; they do not directly test the processes underlying the age associations.

The present study aims to systematically characterize the age-related patterns of visual attentional networks in deaf and hearing individuals from 6 to 19 years of age, as well as to identify between-group differences across four age bands. We recruited 525 participants (262 males, 263 females), including 200 deaf individuals and 325 hearing individuals. Age-related patterns of attentional functioning were assessed with the classic ANT and modeled by comparing a series of regression models of age. Three primary hypotheses were examined in the present study. First, based on evidence that the diffusion decision model separates processing efficiency from the speed–accuracy trade-off, and that attentional redistribution in deaf participants has been associated with differences in estimated drift rate rather than boundary separation, we hypothesized that deaf and hearing participants would differ in DDM parameters during attention-network performance. These differences were expected to manifest in drift rate, rather than in boundary separation or non-decision time. Second, given prior evidence that the three attention networks show different age-related patterns in hearing individuals, we hypothesized that the cross-sectional associations between age and ANT scores would differ between groups. These differences were predicted to emerge as nonlinear age functions, particularly for executive control and orienting, in hearing participants, with distinct functional forms in deaf participants. Third, building on previous research showing that adolescence is a major period of neurocognitive reorganization and that group differences in attention may widen with age, we hypothesized that between-group contrasts would be smaller during childhood and larger in adolescence. Specifically, deaf participants were predicted to show larger executive-control conflict costs and smaller spatial-cue benefits than hearing participants during adolescence.

## 2. Materials and Methods

### 2.1. Participants

The sample included 525 participants (262 male and 263 female participants), including 200 deaf participants and 325 hearing participants. All participants were recruited from multiple special-education and mainstream schools in China (see Table 1 for detailed demographic information). All participants were right-handed, had normal or corrected-to-normal vision, and reported no history of neurological or psychiatric disorders. The etiologies of deafness were classified as exposure to ototoxic drugs (*n* = 176) or genetic causes (*n* = 24). Prelingual deafness was defined as hearing loss present at birth or acquired before the establishment of spoken language, with onset before age 3 years (Jallu et al., 2019). All deaf participants met this criterion and had severe-to-profound bilateral hearing loss, with a documented onset at *M* = 1.59 years (*SD* = 1.24, range = 0.35–2.83 years). Eligibility was based on the official Medical Certification of Disability issued by the China Disabled Persons’ Federation. This certification requires a clinical audiological evaluation at a designated tertiary hospital and verification of severe-to-profound bilateral hearing loss (>90 dB HL across speech frequencies). No deaf participant had ever used a hearing aid or cochlear implant. All deaf participants were enrolled in special-education schools serving economically disadvantaged regions of China. Given regional, economic, and family circumstances, none had been exposed to Chinese Sign Language (CSL) before entering primary school. All began learning CSL systematically only after entering school, and school entry was often delayed. The hearing group comprised participants who spoke only Chinese and had no prior exposure to CSL. We conducted a sensitivity power analysis using Monte Carlo simulation with 2000 replications per condition for the two-term Group × Age likelihood-ratio tests (*df* = 2, *α* = 0.05). With the final sample of 525 participants, the design had 80% power to detect interaction effects of *f*^2^ = 0.018, corresponding to an incremental explained variance of approximately 1.8%. This analysis indicated that the sample size provided adequate sensitivity to the interaction effects examined. Participants or their legal guardians provided written informed consent, and participants received financial compensation. The institutional ethics committee approved the study (approval number omitted for double-blind review). The study was conducted in accordance with the Declaration of Helsinki.

### 2.2. Materials and Procedure

**Figure 1:**
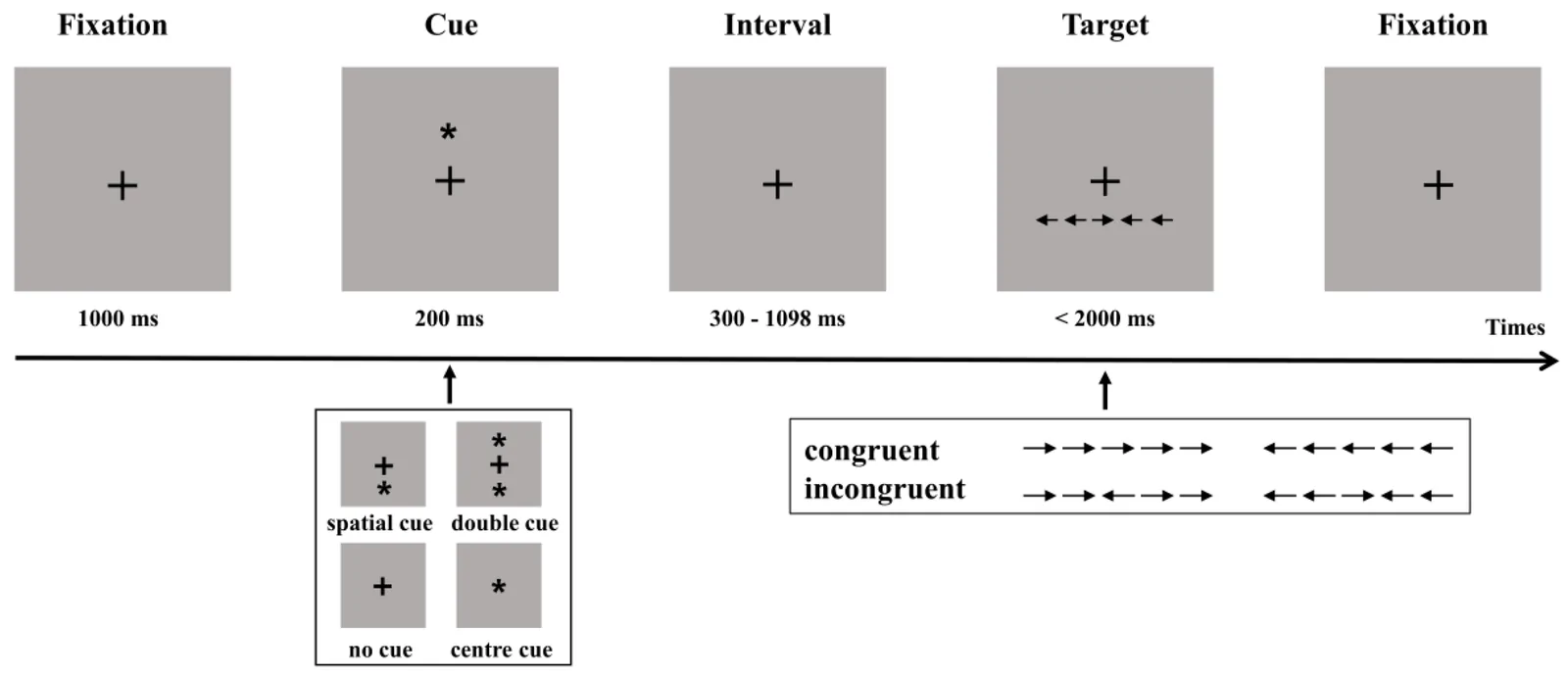
An example trial in the Attention Network Test procedure. The asterisk indicates the cue, the plus sign indicates the fixation point, and the arrows represent the stimuli.

We used the ANT (see Figure 1) developed by Rueda, Fan, and colleagues (Fan et al., 2002). A fixation cross remained at the center of the screen, and one of four cue conditions preceded the target: a spatial cue appeared above or below fixation; a center cue appeared at fixation; a double cue appeared at both locations; and the no-cue condition provided no warning signal. Target arrays consisted of a central arrow flanked by four arrows, were presented 1.06° above or below fixation, and were either congruent or incongruent. Each arrow subtended 0.58°, adjacent stimuli were separated by 0.06°, and the complete array subtended 3.27°. Participants reported the direction of the central arrow by pressing the left or right response key while ignoring the flankers. Each block included all combinations of the two flanker types and four cue conditions. Participants completed 96 trials across two blocks, with approximately 12 trials per cue-by-flanker cell, 48 trials per flanker condition, and 24 trials per cue condition before exclusions. E-Prime 2.0 controlled stimulus presentation and response recording. We used the adult version of the arrow ANT across the full age range; comparable flanker stimuli have been administered reliably to children as young as 5 years (Ridderinkhof et al., 1997).

### 2.3. Statistical Analyses

#### 2.3.1. ANT Components

For each participant and condition, mean response time (RT) was calculated from correct trials after excluding trials with RTs below 100 ms or above 2000 ms. This criterion excluded 185 trials with RTs below 100 ms, which were treated as anticipatory responses; no trial exceeded 2000 ms. Incorrect responses and omissions were tallied separately, and accuracy was defined as the proportion of correct responses. We expressed ANT scores as proportional RT contrasts to facilitate comparisons across participants with different response speeds in the 6–19-year age range. If RTs in both conditions are proportionally longer, the same relative task effect produces a larger absolute difference in milliseconds. Dividing the conventional ANT condition contrast (Fan et al., 2002) by the participant’s RT in the corresponding reference condition expresses the effect relative to that RT scale. The reference conditions were congruent trials for executive control, spatial cues for orienting, and double cues for alerting. These scores should therefore be interpreted as analysis-specific proportional measures. Higher executive-control scores indicate greater relative conflict costs, whereas higher orienting and alerting scores indicate greater relative cue benefits. To assess sensitivity to the scoring method, we also examined the continuous Group × Age interactions using conventional raw RT differences in milliseconds.

$$\mathrm{Executive}\;\mathrm{control}=\;\frac{{\mathrm{RT}}_{\mathrm{incongruent}\;}-\;{\mathrm{RT}}_{\mathrm{congruent}\;}}{{\mathrm{RT}}_{\mathrm{congruent}}}$$

$$\mathrm{Orienting}\;=\;\frac{{\mathrm{RT}}_{\mathrm{center}\;\mathrm{cue}\;}-\;{\mathrm{RT}}_{\mathrm{Spatial}\;\mathrm{cue}\;}}{{\mathrm{RT}}_{\mathrm{Spatial}\;\mathrm{cue}}}$$

$$\mathrm{Alerting}\;=\;\frac{{\mathrm{RT}}_{\mathrm{no}\;\mathrm{cue}}-\;{\mathrm{RT}}_{\mathrm{double}\;\mathrm{cue}\;}}{{\mathrm{RT}}_{\mathrm{double}\;\mathrm{cue}}}$$

#### 2.3.2. EZ-Diffusion Parameter Estimation and Analysis

The EZ-diffusion model (Wagenmakers et al., 2007), a closed-form approximation to the drift-diffusion model (Ratcliff & Rouder, 1998), estimates three decision parameters from response accuracy and the mean and variance of correct-trial RTs: drift rate (v), which indexes the efficiency of evidence accumulation; boundary separation (a), which indexes response caution; and non-decision time (Ter), which captures processes outside the decision process, such as stimulus encoding and motor execution. Parameters were estimated for each participant and condition after applying the same 100–2000 ms RT filter described above. Each cell was required to contain at least five analyzed trials, at least two correct responses, and positive variance in correct-trial RTs; accuracy values of 0, 0.5, or 1 were adjusted by 1/(2N) following Wagenmakers et al. (2007). We applied no additional winsorization or post-estimation trimming based on EZ parameter magnitudes. Cell screening was limited to the prespecified requirements for trial count, correct-response count, variance, and finite parameter estimates. All observed participant-by-condition cells produced finite parameter estimates. Correct-response counts per cell ranged from 9 to 48; the 21 cells with accuracy below 0.5 yielded negative drift-rate estimates, and no estimate of boundary separation or non-decision time was negative. Median retained trial counts ranged from 39 to 40 per flanker condition and 20 trials per cue condition (Supplementary Table S1). All 525 participants were retained for every network. For each network and parameter, we fit a 2 (Condition) × 2 (Group: deaf vs. hearing) linear mixed-effects model with a by-participant random intercept and restricted maximum likelihood estimation (nlme package, version 3.1-163; Pinheiro et al., 2023). The *p*-values for the nine Condition × Group tests (three networks × three parameters) were adjusted using the Holm method. Separate sensitivity models included Raven IQ and sex as covariates in separate analyses. As complementary evidence, we computed a JZS Bayes factor for each parameter using per-participant condition-difference scores, the default Cauchy prior (Rouder et al., 2009), and the BayesFactor package (Morey & Rouder, 2024). All analyses were conducted in R (Version 4.3.0; R Core Team, 2023). As a robustness check, we repeated the EZ analyses using lower RT cutoffs of 150 and 200 ms while retaining the 2000-ms upper cutoff. The pattern of Condition × Group interactions remained unchanged after Holm adjustment.

#### 2.3.3. Age-Related Pattern Analysis

Age-related functions were estimated using fixed-effects generalized least-squares (GLS) models with the nlme package (Pinheiro et al., 2023). Because each participant contributed one cross-sectional observation, no participant-level random effect was included. The fitted age functions characterize cross-sectional associations between age and ANT performance and do not estimate within-person developmental change. Age was the primary predictor rather than a nuisance covariate. Age was mean-centered before polynomial terms were formed, and the centered age terms and their interactions with Group were used to test between-group differences in the combined models. Within each group, we compared five candidate age specifications using maximum likelihood:

1. Null model: Y = intercept + error;
2. Logarithmic age model: Y = intercept + log(age) + error;
3. Linear age model: Y = intercept + age + error;
4. Quadratic age model: Y = intercept + age^2^ + age + error;
5. Cubic age model: Y = intercept + age^3^ + age^2^ + age + error.

**Figure 2:**
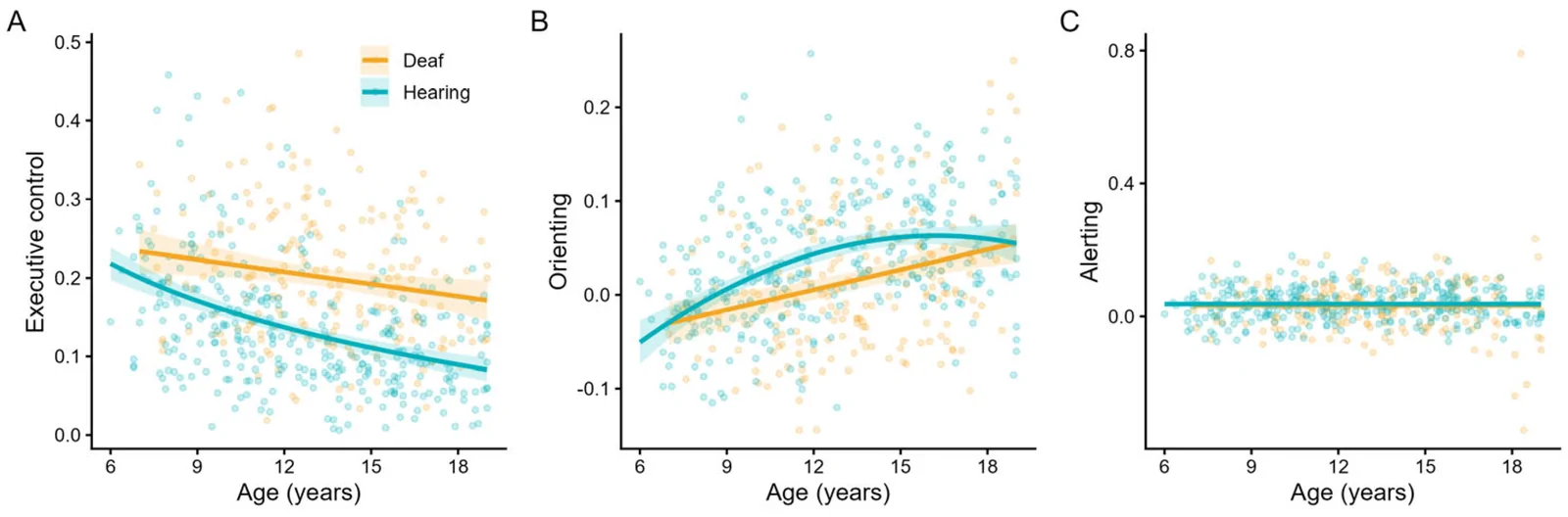
Cross−sectional age−related patterns of the three attentional components: (**A**) executive control; (**B**) orienting alerting; and (**C**) alerting. Lines represent the BIC-selected generalized least-squares age functions fitted separately within each group, and shaded areas represent 95% confidence intervals. The separately fitted curves are descriptive. The combined Group × Age interaction was not significant for executive control (*p*_Holm_ = 0.166) or alerting (*p*_Holm_ = 0.917), but was significant for orienting (*p*_Holm_ = 0.002).

BIC (Schwarz, 1978) was the primary model-selection criterion, and the model with the smallest BIC was retained. AIC (Akaike, 1974) and nested likelihood-ratio tests (LRTs) were treated as supplementary criteria. Because BIC applies a stronger penalty for model complexity, it helps limit overfitting by higher-order terms in cross-sectional age data (Fjell et al., 2010). The BIC-selected model for each group was plotted with its 95% confidence interval in Figure 2. Between-group differences in age-related functions were tested in the full sample by comparing an additive model with a fully interactive model: Additive model (Y = intercept + Group + Age + Age^2^ + error); Fully interactive model (Y = intercept + Group × (Age + Age^2^) + error). The models were compared using an LRT. The two additional interaction terms, Group × centered Age and Group × centered Age^2^, account for the reported two degrees of freedom. The combined model comparison was the primary test of whether the age-related functions differed between groups. Holm correction was applied across the three network interaction tests. Sensitivity models added sex or Raven IQ as a covariate separately and repeated the analyses using raw RT difference scores instead of normalized ANT scores.

#### 2.3.4. Age-Band Analyses

To complement the primary continuous-age models, we grouped participants into four a priori age bands to identify the age ranges in which between-group differences were observed: childhood (6–9 years; deaf, *n* = 27; hearing, *n* = 92), early adolescence (10–14 years; deaf, *n* = 108; hearing, *n* = 130), middle adolescence (15–17 years; deaf, *n* = 54; hearing, *n* = 81), and late adolescence (18–19 years; deaf, *n* = 11; hearing, *n* = 22), following standard developmental-stage conventions (Sawyer et al., 2018). We analyzed network scores using two-way ANOVAs with Group (deaf, hearing) and Age Band as between-participants factors, Type III sums of squares, and partial eta squared as the effect-size measure. The *p*-values for the three Group × Age Band interaction tests were adjusted across networks using the Holm method, and those for the four within-network group contrasts were adjusted using the Bonferroni method. Accordingly, age entered these analyses only through the prespecified Age Band factor. Separate sensitivity models added sex and Raven IQ as covariates in separate analyses.

## 3. Results

### 3.1. Overall Behavioral Performance

Table 2 summarizes reaction times and accuracy for the deaf and hearing groups under each task condition. Both groups performed the task accurately: hearing participants achieved accuracy at or above 92% in all conditions. Deaf participants showed lower accuracy on incongruent trials (81.2%) despite correct-trial RTs that were comparable to those of hearing participants. Descriptive analyses confirmed the three expected attentional effects in both groups: response times were shorter for congruent than incongruent trials (executive/flanker effect), for spatially cued than center-cued trials (orienting effect), and for double-cued than no-cue trials (alerting effect; see Table 2). These patterns indicate that the task elicited the intended attentional effects in both the deaf and hearing samples.

### 3.2. EZ-Diffusion Modeling Results

#### 3.2.1. Drift Rate

For executive control, a 2 (Flanker: congruent vs. incongruent) × 2 (Group) model revealed a large main effect of Flanker, *F*(1, 523) = 491.65, *p* < .001, and no main effect of Group, *F*(1, 523) = 0.90, *p* = .344. The Flanker × Group interaction was significant and survived Holm correction across the nine diffusion interaction tests, *F*(1, 523) = 94.97, *p* < .001, η^2^*_p_* = 0.154, with decisive Bayesian support (BF10 > 1000). Incongruent trials produced lower drift rates than congruent trials in both groups, but the reduction was greater among deaf participants (*v*_congruent_ = 0.289, *v*_incongruent_ = 0.171) than among hearing participants (*v*_congruent_ = 0.282, *v*_incongruent_ = 0.230, see Table 2), corresponding to a larger conflict-related reduction in estimated drift rate in the deaf group. For orienting, the Cue (spatial vs. center) × Group interaction on drift rate was not significant, *F*(1, 523) = 0.26, *p* = .613, η^2^*_p_* < 0.001, with moderate Bayesian support for the null (BF01 = 8.85); drift rates changed little between cue conditions in either group (deaf: *v*_spatial_ = 0.209, *v*_center_ = 0.209; hearing: *v*_spatial_ = 0.241, *v*_center_ = 0.239). The main effect of Cue was not significant, *F*(1, 523) = 0.01, *p* = .918, whereas the main effect of Group was, *F*(1, 523) = 21.35, *p* < .001, reflecting globally lower drift rates in the deaf group. For alerting, the Cue (double vs. no cue) × Group interaction on drift rate was not significant, *F*(1, 523) = 1.90, *p* = .168, η^2^*_p_* = 0.004, *p*_Holm_ = 0.505, with moderate Bayesian support for the null (BF01 = 3.98). Relative to the no-cue condition, double cues did not reliably elevate drift rate in either group (hearing: *v*_double_ = 0.244, *v*_no-cue_ = 0.235; deaf: *v*_double_ = 0.210, *v*_no-cue_ = 0.209). The main effect of Cue was not significant, *F*(1, 523) = 0.07, *p* = .787, whereas the main effect of Group was, *F*(1, 523) = 24.94, *p* < .001.

#### 3.2.2. Non-Decision Time

For executive control, the Flanker × Group interaction on non-decision time was significant and survived Holm correction, *F*(1, 523) = 18.07, *p* < .001, *p*_Holm_ < 0.001, η^2^*_p_* = 0.033, with decisive Bayesian support (BF10 = 574.64). Incongruent trials lengthened non-decision time in both groups, more so among deaf participants (*Ter*_congruent_ = 487 ms, *Ter*_incongruent_ = 606 ms) than among hearing participants (*Ter*_congruent_ = 511 ms, *Ter*_incongruent_ = 599 ms, see Table 2). For orienting, the Cue × Group interaction was not significant, *F*(1, 523) = 3.84, *p* = .051, η^2^*_p_* = 0.007, with anecdotal Bayesian support for the null (BF01 = 1.56); the main effect of Cue was significant, *F*(1, 523) = 13.01, *p* < .001, and that of Group was not, *F*(1, 523) = 2.10, *p* = .148. Alerting patterned similarly: the Cue × Group interaction was not significant, *F*(1, 523) = 2.50, *p* = .114, η^2^*_p_* = 0.005, with anecdotal Bayesian support for the null (BF01 = 2.98), the main effect of Cue was significant, *F*(1, 523) = 12.48, *p* < .001, and that of Group was not, *F*(1, 523) = 3.32, *p* = .069.

#### 3.2.3. Boundary Separation

For executive control, the Flanker × Group interaction on boundary separation was nominally significant, *F*(1, 523) = 5.77, *p* = .017, η^2^*_p_* = 0.011, but did not survive Holm correction (*p*_Holm_ = 0.116), and the Bayesian evidence was only anecdotal (BF10 = 1.63); it is therefore not interpreted further. The main effect of Flanker was significant, *F*(1, 523) = 44.14, *p* < .001, and the main effect of Group was significant, *F*(1, 523) = 18.05, *p* < .001, with lower estimated boundary separation in deaf participants (Table 2). For orienting, the Cue × Group interaction was not significant, *F*(1, 523) = 0.73, *p* = .393, η^2^*_p_* = 0.001, with moderate Bayesian support for the null (BF01 = 7.02), and the main effect of Group was significant, *F*(1, 523) = 28.46, *p* < .001, with boundaries being lower in the deaf group. For alerting, the Cue × Group interaction was not significant, *F*(1, 523) = 3.15, *p* = .077, η^2^*_p_* = 0.006, with anecdotal Bayesian support for the null (BF01 = 2.18), and the main effect of Group was significant, *F*(1, 523) = 36.70, *p* < .001, again with lower boundaries among deaf participants.

Taken together, the EZ analyses showed larger conflict-related differences in estimated drift rate and non-decision time among deaf participants, with both Condition × Group interactions surviving Holm correction. The corresponding interaction for boundary separation did not survive correction. For orienting and alerting, no Condition × Group interaction was reliable for any parameter; the observed group differences reflected global differences in drift rate and boundary separation rather than cue-related modulation. Sensitivity models including Raven IQ or sex as covariates left all nine Condition × Group interactions essentially unchanged, with the same Holm-adjusted conclusions (Supplementary Table S2).

### 3.3. Age-Related Patterns of the ANT Components

Within-group BIC comparisons selected a linear model for deaf participants and a logarithmic model for hearing participants (Table 3). These models described cross-sectional associations in which conflict costs were lower among older participants in both groups, with a steeper and decelerating association in the hearing group. However, the primary combined Group × Age likelihood-ratio test was not significant, *L.Ratio* = 4.98, *df* = 2, *p* = .083, *p*_Holm_ = 0.166. Thus, the separately selected linear and logarithmic forms should be interpreted as descriptive summaries and do not provide evidence of statistically different age-related functions between groups. The same conclusion was obtained after sex adjustment, *L.Ratio* = 4.77, *p* = .092, and adjustment for Raven IQ, *L.Ratio* = 5.19, *p* = .075. When raw RT difference scores were used instead of normalized scores, the interaction was nominally significant, *L.Ratio* = 6.47, *df* = 2, *p* = .039, but did not survive Holm correction across the three networks (*p*_Holm_ = 0.118). This scoring-dependent result is reported as a sensitivity analysis and is not treated as definitive evidence of different age-related functions. For orienting, BIC selected a linear function in deaf participants and a decelerating quadratic function in hearing participants (Table 4); orienting scores were higher at older ages in both groups, with a flatter fitted age association at older ages in the hearing group. The combined interaction was significant, *L.Ratio* = 14.31, *df* = 2, *p* < .001, *p*_Holm_ = 0.002, and remained significant after sex adjustment, *L.Ratio* = 14.54, *p* < .001 and after adjustment for Raven IQ, *L.Ratio* = 14.33, *p* < .001. For alerting, BIC retained the null model in both groups (Table 5; a weak quadratic trend in the hearing group was preferred by AIC but not BIC), and the combined interaction was not significant, *L.Ratio* = 0.17, *df* = 2, *p* = .917 and remained so after sex (*L.Ratio* = 0.13, *p* = .939) and Raven-IQ (*L.Ratio* = 0.19, *p* = .910) adjustment. Scoring sensitivity qualified these results (Supplementary Table S3). With raw RT differences, the executive-control interaction was nominally significant (uncorrected p = 0.039) but did not survive Holm correction (*p*_Holm_ = 0.118), the orienting interaction was not, *L.Ratio* = 1.09, *df* = 2, *p* = .581; raw alerting remained nonsignificant (*p* = .654). Thus, the orienting interaction was reliable only under normalized scoring, and no raw-score interaction survived Holm correction; alerting was robustly null across scoring methods. Complete parameter estimates for the six combined models and the likelihood-ratio details are provided in Supplementary Tables S4 and S5; parameter estimates for the BIC-selected within-group models are presented in Table 6.

### 3.4. Differences in ANT Components Across Four Age Bands

**Figure 3:**
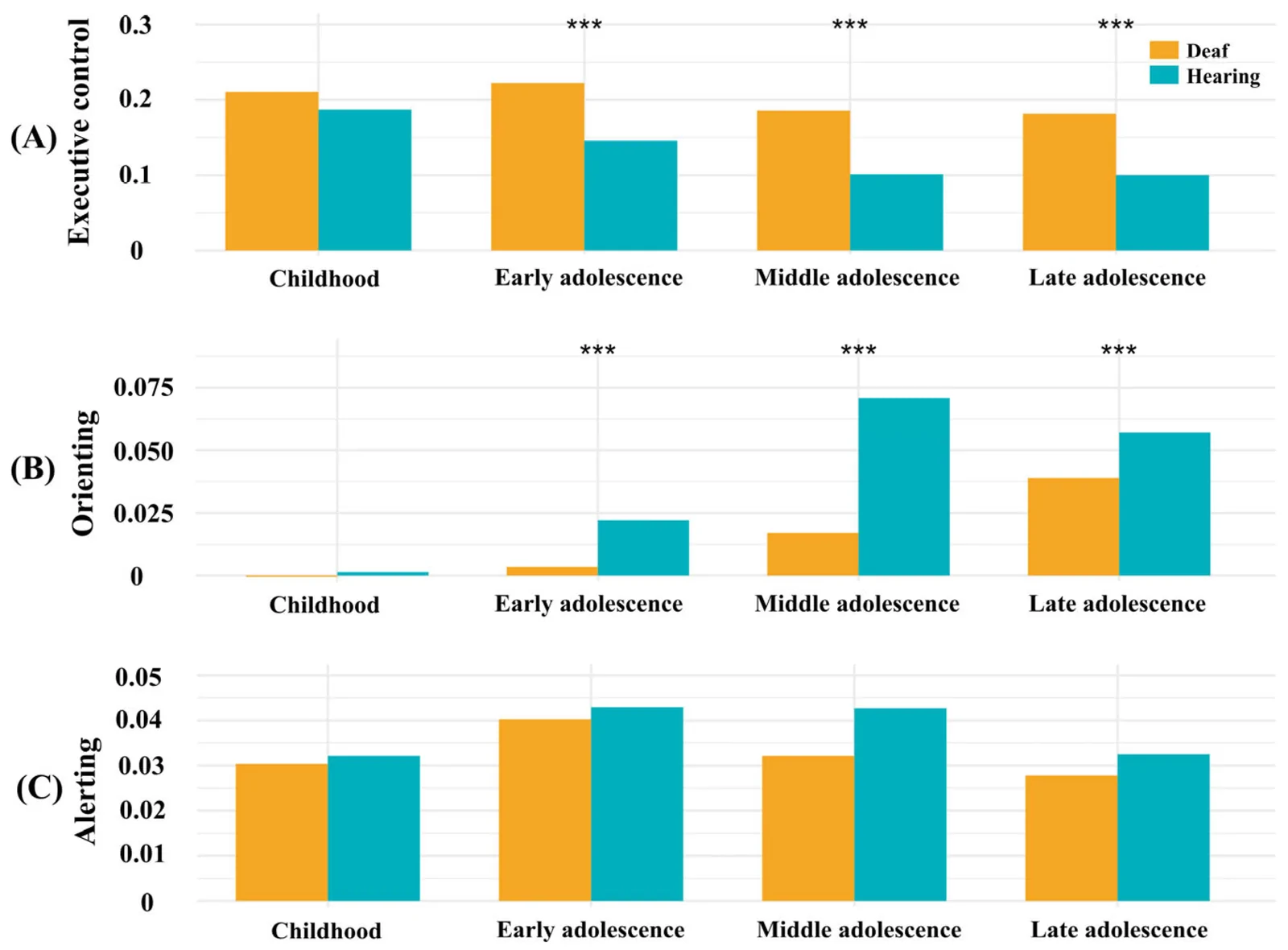
ANT component scores across four age bands for (**A**) executive control; (**B**) orienting; and (**C**) alerting. The age bands were childhood (6–9 years), early adolescence (10–14 years), middle adolescence (15–17 years), and late adolescence (18–19 years). Asterisks indicate Bonferroni-adjusted group comparisons within individual age bands (*** *p* < .001), rather than the overall Group × Age Band interactions. The Group × Age Band interaction was not significant for executive control (*p*_Holm_ = 0.060) or alerting (*p*_Holm_ = 0.975), but was significant for orienting (*p*_Holm_ < 0.001).

Figure 3 displays the three network scores by age band and group. For executive control (Figure 3A), conflict scores were highest in childhood and were lower in the older age bands (main effect of Age Band, *F*(3, 517) = 8.83, *p* < .001, η^2^*_p_* = 0.049), and the deaf group showed larger conflict costs overall (main effect of Group, *F*(1, 517) = 48.40, *p* < .001, η^2^*_p_* = 0.086). The Group × Age Band interaction was nominal, *F*(3, 517) = 3.00, *p* = .030, η^2^*_p_* = 0.017, but did not survive Holm correction across the three networks (*p*_Holm_ = 0.060). Bonferroni-adjusted contrasts showed larger conflict costs in deaf participants during early adolescence (*p* < .001), middle adolescence (*p* < .001), and late adolescence (*p* < .001), but not childhood (*p* = .572). Sex adjustment yielded similar estimates; its interaction was *F*(3, 516) = 2.94, *p* = .033, *p*_Holm_ = 0.066, and adjustment for Raven IQ likewise, *F*(3, 516) = 3.10, *p* = .026, *p*_Holm_ = 0.053. For orienting (Figure 3B), scores were higher in the older age bands (main effect of Age Band, *F*(3, 517) = 18.27, *p* < .001, η^2^*_p_* = 0.096), with no main effect of Group, *F*(1, 517) = 0.01, *p* = .944. The Group × Age Band interaction was significant and survived Holm correction, *F*(3, 517) = 10.87, *p* < .001, η^2^*_p_* = 0.059, *p*_Holm_ < 0.001. The spatial-cue benefit was smaller in deaf participants during early and middle adolescence (both Bonferroni-adjusted *p* < .001), comparable in childhood (*p* = 1.000), and smaller in deaf participants during late adolescence (*p* < .001). The late-adolescent cells were small (11 deaf and 22 hearing participants), so the result requires replication. For alerting (Figure 3C), no main effect or interaction was significant (Group: *F*(1, 517) = 0.47, *p* = .493; Age Band: *F*(3, 517) = 1.53, *p* = .207; Group × Age Band: *F*(3, 517) = 0.07, *p* = .975). These categorical analyses localize observed contrasts but are complementary to, and weaker than, the continuous-age analyses. In particular, the adolescent executive-control contrasts should not be interpreted as evidence that an individual-level transition occurs at adolescence. Sex and Raven-IQ-adjusted results were substantively similar (Supplementary Table S6).

## 4. Discussion

In this cross-sectional study of 525 participants aged 6–19 years, group differences were network-specific rather than consistent with a generalized impairment or compensatory advantage. Alerting showed no reliable group or age-related differences. For orienting, both the Group × Age and Group × Age Band interactions survived Holm correction, although the continuous interaction was found only with normalized scores and was not reproduced using raw RT differences. Deaf participants also showed higher executive-control costs in the adolescent age bands, but neither executive-control interaction survived correction; the different within-group age functions should therefore be treated as descriptive. Accordingly, all three hypotheses received partial support. Hypothesis 1 was supported by the larger conflict-related reduction in estimated drift rate in deaf participants, but the non-decision-time interaction and overall boundary-separation differences showed that parameter differences were not confined to drift rate. Hypothesis 2 was supported only for orienting under normalized scoring. Hypothesis 3 was supported by the predicted age-band contrast pattern, although only orienting provided corrected evidence that group differences varied across age bands.

### 4.1. Executive Control Network

The present study found localized group contrasts in executive-control performance across the adolescent age bands, but not in childhood. In the hearing group, the separately fitted logarithmic model described lower conflict scores among older participants, with a steeper negative age association at younger ages and a flatter association at older ages. This cross-sectional pattern is consistent with prior research (Boen et al., 2021; Davidson et al., 2006; Diamond, 2013; Waszak et al., 2010). Although synaptic pruning and cortical reorganization have been discussed in relation to executive-control development (Fair et al., 2007; Konrad et al., 2005), the present data do not measure these processes or establish within-person developmental change. For the deaf group, the separately fitted model indicated an approximately linear association between age and conflict scores, with older age associated with lower conflict scores. However, the combined Group × Age interaction was not statistically significant; the contrast between the logarithmic and linear fits should therefore be interpreted as descriptive rather than as evidence of statistically distinct age functions. Although the groups performed comparably during childhood, deaf participants showed larger executive-control costs during early, middle, and late adolescence.

The EZ-diffusion results provided further insight into the processes underlying this executive-control difference. Relative to congruent trials, incongruent trials were associated with a larger reduction in drift rate and a larger increase in non-decision time among deaf participants than among hearing participants. Non-decision time (Ter) captures processes outside evidence accumulation, including stimulus encoding and response execution. However, the EZ analysis cannot identify which component accounts for a group difference in this parameter. Together, these effects suggest that conflict was associated with both less efficient evidence accumulation and a larger aggregate non-decision component in deaf participants, although the latter effect cannot be attributed to a specific subprocess. Deaf participants also showed lower boundary separation overall, consistent with generally less cautious responding. The Flanker × Group interaction for boundary separation did not survive Holm correction, suggesting broadly comparable conflict-related changes in response caution between groups. Contrary to our initial hypothesis that group differences would be confined to drift rate, a Flanker × Group interaction was also observed for non-decision time, suggesting that conflict-related processes outside evidence accumulation may also differ between groups. Thus, the executive-control difference appears to involve conflict-related changes in evidence accumulation and aggregate non-decision processes rather than group differences in conflict-specific adjustments of response caution.

Several mechanisms could account for the executive-control difference observed during adolescence, but the present data cannot distinguish among these possibilities. The auditory scaffolding hypothesis and auditory connectome model suggest that early auditory deprivation may alter connectivity between auditory regions and higher-order cognitive-control systems, including the PFC (Merchan et al., 2022; Okely et al., 2021). As demands on endogenous control increase during adolescence, differences in these systems may become more evident. Language and communication experience may also contribute to the observed executive-control pattern. Executive-control performance may also be shaped by vocabulary, language-mediated working memory, inner speech, and familiarity with task instructions and response rules (Botting et al., 2017; Hall et al., 2018; Jones et al., 2020; Marshall et al., 2015; Merchan et al., 2022). Sign-language experience may also redistribute visual attentional resources, so communication history may be relevant to orienting as well as to executive control (Stoll & Dye, 2019). In this sample, all deaf participants first encountered Chinese Sign Language (CSL) after entering primary school, whereas hearing participants had no prior exposure to CSL. For deaf participants, the documented age at hearing-loss onset and years since first CSL exposure offer descriptive information about auditory and language-access histories, but they do not measure CSL proficiency, vocabulary, or the quality of communication experience. We also did not assess the communication mode used at home or the age at which participants first accessed a fully accessible language. Therefore, the adolescent executive-control differences observed in this study could reflect differences in language experience, communication history, educational context, hearing status, or some combination of these factors. The data therefore do not allow us to determine which explanation best accounts for the observed pattern.

### 4.2. Orienting Network

The age-related group differences observed in the orienting network should therefore be interpreted cautiously with respect to the enhancement hypothesis. The fitted cross-sectional age association was quadratic in hearing participants, with a flatter curve at older ages, and approximately linear in deaf participants. Although spatial-cue benefits were comparable during childhood, deaf participants showed smaller benefits during early and middle adolescence, as reflected in lower ANT orienting scores. This pattern may appear inconsistent with the traditional enhancement hypothesis, which holds that early auditory deprivation may be accompanied by cross-modal reorganization and enhanced visuospatial orienting, particularly in the exogenous capture of peripheral stimuli (Bavelier et al., 2006; Gioiosa Maurno et al., 2024; Lore & Song, 1991; Stevens & Neville, 2006). However, this apparent discrepancy may reflect the cognitive demands imposed by the ANT. The orienting index is calculated as the difference between the center-cue and spatial-cue conditions and therefore reflects not only peripheral detection but also disengagement from fixation, attentional shifting, and re-engagement at the target location (Daza & Phillips-Silver, 2013). Accordingly, a smaller orienting score indicates a smaller proportional benefit from a spatial cue under this ANT contrast; by itself, it does not demonstrate a generalized visuospatial orienting impairment or a deficit in disengagement.

The EZ-diffusion results further clarified the distinction between overall decision characteristics and cue-specific orienting effects. Deaf participants showed lower drift rates and boundary separation across cue conditions, suggesting less efficient evidence accumulation and a less conservative response criterion. However, none of the diffusion parameters showed a reliable Cue × Group interaction. Spatial cues did not differentially affect drift rate, non-decision time, or boundary separation in the two groups. Thus, the age-related difference in the proportional spatial-cue benefit was not accompanied by a reliable group difference in the decision processes specifically engaged by spatial versus central cues. Instead, the diffusion results point to broader group differences in decision efficiency and response caution, whereas cue-related modulation was broadly similar between groups.

The ANT may therefore capture a more complex form of orienting than tasks focused primarily on exogenous responses to peripheral stimuli. Efficient performance may depend on coordinated activity across higher-order frontoparietal regions, including the temporoparietal junction and superior parietal lobule (Petersen & Posner, 2012). Moreover, compensatory advantages in peripheral vision are not necessarily stable across developmental stages or task contexts. Codina et al. (2011) found that such advantages may emerge only during middle-to-late adolescence or under specific task demands. Accordingly, these results do not support either a generalized deficit or a uniform compensatory enhancement of visual orienting. Instead, group differences varied across attentional operations and age bands, a pattern consistent with the integrative hypothesis proposed by M. W. Dye and Bavelier (2010). Language and communication experience may also contribute to the orienting findings. Although sign-language experience has been reported to influence the allocation of visual-attentional resources (Stoll & Dye, 2019), we did not measure sign-language proficiency, communication history, or related visual-attentional strategies. Consequently, the observed orienting differences cannot be uniquely attributed to auditory deprivation, sign-language experience, or any other single factor.

### 4.3. Alerting Network

Under the primary BIC criterion, the null model was selected for alerting in both groups, providing no reliable evidence of an association between age and alerting scores in this sample. No significant group difference was detected. Although AIC favored a quadratic age function in hearing participants, this result depended on the model-selection criterion and should be interpreted cautiously. These cross-sectional findings do not establish within-person stability in alerting over time.

The EZ-diffusion results provided further insight into the processes underlying alerting. Deaf participants showed lower drift rates and boundary separation across alerting conditions, suggesting less efficient evidence accumulation and a less conservative response criterion. The Cue × Group interaction for drift rate was not significant, and the corresponding Bayes factor provided moderate evidence for the absence of this interaction, indicating that warning cues affected evidence accumulation similarly in the two groups. Moreover, Cue × Group interactions were not significant for non-decision time or boundary separation, indicating broadly similar cue-related modulation of these processes. No reliable behavioral group differences were observed in any of the four age bands, indicating that no detectable group difference in phasic alerting emerged across the age range studied. Taken together, these findings suggest that phasic alerting elicited by exogenous warning cues is relatively preserved in deaf participants (Ma et al., 2023). This relative preservation contrasts with the group differences observed in executive control and complex spatial orienting, supporting the view that group differences vary across attentional networks. This network-specific pattern is compatible with the integrative perspective discussed by M. W. Dye and Bavelier (2010). However, the present behavioral comparisons do not establish whether cross-modal plasticity, language experience, or educational context accounts for this pattern.

### 4.4. Implications and Limitations

The main contribution of this study is to extend previous attention-network research in deaf children to a sample spanning ages 6–19 and to combine cross-sectional ANT analyses with complementary estimates of decision processes. Building on childhood ANT research (Daza & Phillips-Silver, 2013) and diffusion-model research on visual attention in adults (Stoll & Dye, 2019), this combination addresses two related questions within the same sample: how attention-network scores differ between groups across age, and how conflict and cue manipulations are associated with group differences in estimated decision parameters. The added value is illustrated by executive control. Although mean correct-trial reaction times on incongruent trials were similar between groups, accuracy was lower among deaf participants. By considering accuracy together with reaction-time means and variances, the EZ analyses identified larger conflict-related reductions in estimated drift rate and increases in estimated non-decision time in the deaf group, with both interactions surviving Holm correction. The boundary-separation interaction did not survive correction, despite lower overall boundaries in deaf participants. For orienting and alerting, overall parameter differences were observed without reliable cue-by-group interactions. These findings help distinguish behavioral conflict costs and cue benefits from overall decision characteristics and condition-related parameter differences. The EZ estimates provide interpretations conditional on the model assumptions; because associations between age and the EZ parameters were not directly tested, they do not establish the mechanisms underlying the ANT age patterns.

This study has several limitations that should be considered. First, the cross-sectional design cannot rule out cohort effects or capture within-person change; longitudinal studies are therefore needed to trace the development of attention networks within individuals over time. Second, the design cannot disentangle auditory deprivation from other between-group differences. The groups differed modestly in age, sex composition, Raven IQ, and educational level. Age was modeled directly, and separate adjustments for sex and Raven IQ yielded similar results. However, CSL proficiency, vocabulary, home communication mode, and age at first access to a fully accessible language were not assessed. Years of CSL exposure do not establish proficiency, and first CSL exposure upon school entry does not identify when consistent access to an accessible language began. Missing home communication data limit our understanding of everyday language access, while absent vocabulary measures prevent assessment of language–executive-control associations (Jones et al., 2020). We therefore cannot distinguish associations with current language ability from those with earlier language access. These unmeasured differences may contribute to both group contrasts and cross-sectional age associations. Adjustment for sex or Raven IQ does not resolve these limitations or separate hearing status from educational context. The deaf sample was restricted to participants with prelingual, severe-to-profound bilateral hearing loss, no history of hearing-aid or cochlear-implant use, and either ototoxic or genetic etiologies. All deaf participants had attended special-education schools in economically disadvantaged regions of China and had begun systematic CSL exposure only after entering school. Together with the small late-adolescent age-band cells, these characteristics limit generalizability and call for cautious interpretation. In addition, school-level clustering could not be modeled because school identifiers were unavailable in the de-identified dataset. Shared educational environments may therefore have contributed to the observed between-group contrasts. Future studies should recruit larger, more diverse samples and use longitudinal or carefully matched designs that directly assess language and communication variables while retaining school-level identifiers for multilevel analysis. Such designs would better distinguish associations with hearing status from influences related to language, education, school context, and cohort. Third, the EZ-diffusion estimates were derived from condition-level summary statistics, with a median of only 20 analyzed trials per cue condition, which may limit their precision. We therefore treat these parameters as complementary model-based summaries rather than direct measures of specific cognitive mechanisms. Drift rate does not directly measure inhibitory control or distractor suppression, boundary separation does not establish deliberate strategy use, and non-decision time cannot separate stimulus encoding from response execution. Normalized ANT scores also depend on reference-condition RTs and their measurement error and may not fully remove general processing-speed effects. Because the Group × Age interaction for orienting was significant only with normalized scores, we interpret it as a scoring-dependent difference in the proportional spatial-cue benefit. Finally, the findings rely solely on behavioral indicators and therefore cannot reveal the underlying neural mechanisms. Future work should combine neural measures with direct assessments of language experience to test candidate auditory, language-related, and connectivity mechanisms.

## 5. Conclusions

In summary, this large cross-sectional study found network-specific, age-related differences in visual attention between deaf and hearing participants. Alerting was broadly comparable between groups, whereas executive-control and orienting differences were most evident during adolescence. These results call for a reappraisal of traditional deficit and compensation accounts and are consistent with the integrative hypothesis of attention in a large cross-sectional sample spanning childhood through late adolescence. Together, they indicate that higher-order attention differences between deaf and hearing youth are network-specific and vary across age bands in this cross-sectional sample. However, the study cannot determine the relative contributions of hearing status, language experience, and educational context.

## Figures and Tables

**Table 1:** Demographics and language background information for all participants.

	Deaf (n = 200)	Hearing (n = 325)	*t/χ* ^2^	*p*-Value
Age (years)	13.31 ± 2.89	12.60 ± 3.51	2.49	0.013
Sex (male/female)	115/85	147/178	7.46	0.006
Nonverbal IQ (CRT raw score; 72 items)	50.13 ± 3.69	52.74 ± 6.62	5.80	<0.001
Education level (primary/junior/senior school)	67/106/27	158/116/51	15.77	<0.001
Cause of deafness (Ototoxic drugs/Genetic)	176/24			
Age of deafness (years)	1.59 ± 1.24			
Years of sign-language experience	4.93 ± 2.40			

*Note. CRT denotes the combined Raven’s test. Age of deafness denotes the documented age at onset of hearing loss; all deaf participants met the criterion for prelingual deafness (onset before 3 years). Years of sign-language experience denotes the interval between first exposure to Chinese Sign Language and participation in the present study. All deaf participants first encountered CSL upon entry to primary school.*

**Table 2:** Descriptive statistics for behavioral and EZ-diffusion measures by condition and group (Mean (*SE*)).

Network	Condition	Group	RT (ms)	Accuracy	Drift Rate (*v*)	Boundary Separation (*a*)	Non-Decision Time (*Ter*) (ms)
Executive Control	congruent	Deaf	704.2 (7.1)	0.969 (0.003)	0.289 (0.005)	0.128 (0.002)	486.7 (5.5)
		Hearing	753.1 (7.4)	0.973 (0.003)	0.282 (0.004)	0.137 (0.001)	510.9 (5.5)
	incongruent	Deaf	846.0 (9.6)	0.812 (0.014)	0.171 (0.008)	0.115 (0.002)	605.6 (7.9)
		Hearing	856.4 (8.7)	0.926 (0.005)	0.230 (0.004)	0.130 (0.001)	598.9 (7.2)
Orienting	Center cue	Deaf	763.1 (7.8)	0.886 (0.009)	0.209 (0.006)	0.115 (0.002)	533.9 (6.6)
		Hearing	813.0 (7.7)	0.947 (0.004)	0.239 (0.004)	0.128 (0.001)	560.5 (5.7)
	Spatial cue	Deaf	757.9 (8.3)	0.895 (0.008)	0.209 (0.006)	0.119 (0.002)	514.9 (6.5)
		Hearing	786.6 (8.2)	0.954 (0.004)	0.241 (0.004)	0.131 (0.001)	528.3 (6.4)
Alerting	Double cue	Deaf	756.9 (7.9)	0.891 (0.008)	0.210 (0.006)	0.117 (0.002)	522.8 (6.6)
		Hearing	792.5 (8.0)	0.954 (0.004)	0.244 (0.004)	0.130 (0.001)	539.7 (6.2)
	No cue	Deaf	782.4 (8.3)	0.895 (0.008)	0.209 (0.006)	0.119 (0.002)	540.1 (6.0)
		Hearing	818.9 (8.1)	0.943 (0.004)	0.235 (0.004)	0.128 (0.001)	566.9 (6.1)

*Note. SE = standard error. RT = mean reaction time on correct trials. v = drift rate; a = boundary separation; Ter = non-decision time.*

**Table 3:** Comparison of models predicting executive control in two groups.

Group	Model	Model Number	*df*	*AIC*	*BIC*	*LL*	*Test*	*L.Ratio*	*p*
Deaf	Null model	1	2	−418.24	−411.64	211.12	—	—	—
	Logarithmic age model	2	3	−422.21	−412.31	214.10	1 vs. 2	5.972	.015
	Linear age model *	3	3	−422.64	−412.74	214.32	1 vs. 3	6.401	.011
	Quadratic age model	4	4	−420.82	−407.63	214.41	3 vs. 4	0.182	.670
	Cubic age model	5	5	−419.01	−402.52	214.51	4 vs. 5	0.193	.661
Hearing	Null model	1	2	−712.79	−705.22	358.40	—	—	—
	Logarithmic age model *	2	3	−775.40	−764.05	390.70	1 vs. 2	64.614	<.001
	Linear age model	3	3	−772.96	−761.60	389.48	1 vs. 3	62.165	<.001
	Quadratic age model	4	4	−774.46	−759.33	391.23	3 vs. 4	3.507	.061
	Cubic age model	5	5	−773.05	−754.13	391.52	4 vs. 5	0.585	.444

Note. * indicates the BIC-selected model within each group. BIC was the primary model-selection criterion; AIC and nested likelihood-ratio tests were supplementary. LL = log-likelihood; L. Ratio = likelihood ratio.

**Table 4:** Comparison of models predicting orienting in two groups.

Group	Model	Model Number	*df*	*AIC*	*BIC*	*LL*	*Test*	*L.Ratio*	*p*
Deaf	Null model	1	2	−479.02	−472.42	241.51	*—*	*—*	*—*
	Logarithmic age model	2	3	−491.87	−481.97	248.93	1 vs. 2	14.851	<.001
	Linear age model *	3	3	−494.01	−484.11	250.00	1 vs. 3	16.991	<.001
	Quadratic age model	4	4	−496.16	−482.97	252.08	3 vs. 4	4.153	.042
	Cubic age model	5	5	−499.21	−482.72	254.60	4 vs. 5	5.046	.025
Hearing	Null model	1	2	−847.03	−839.46	425.52	—	—	—
	Logarithmic age model	2	3	−908.10	−896.75	457.05	1 vs. 2	63.072	<.001
	Linear age model	3	3	−902.18	−890.83	454.09	1 vs. 3	57.146	<.001
	Quadratic age model *	4	4	−914.01	−898.87	461.00	3 vs. 4	13.830	<.001
	Cubic age model	5	5	−916.38	−897.46	463.19	4 vs. 5	4.370	.037

Note. * indicates the BIC-selected model within each group. BIC was the primary model-selection criterion; AIC and nested likelihood-ratio tests were supplementary. LL = log-likelihood; L. Ratio = likelihood ratio.

**Table 5:** Comparison of models predicting alerting in two groups.

Group	Model	Model Number	*df*	*AIC*	*BIC*	*LL*	*Test*	*L.Ratio*	*p*
Deaf	Null model *	1	2	−383.45	−376.85	193.72	—	—	—
	Logarithmic age model	2	3	−381.50	−371.61	193.75	1 vs. 2	0.058	.810
	Linear age model	3	3	−381.56	−371.66	193.78	1 vs. 3	0.113	.737
	Quadratic age model	4	4	−380.11	−366.91	194.05	3 vs. 4	0.547	.460
	Cubic age model	5	5	−378.19	−361.70	194.10	4 vs. 5	0.087	.769
Hearing	Null model *	1	2	−1040.60	−1033.04	522.30	—	—	—
	Logarithmic age model	2	3	−1039.69	−1028.34	522.85	1 vs. 2	1.088	.297
	Linear age model	3	3	−1039.11	−1027.76	522.56	1 vs. 3	0.507	.477
	Quadratic age model	4	4	−1044.03	−1028.89	526.01	3 vs. 4	6.915	.009
	Cubic age model	5	5	−1042.23	−1023.31	526.12	4 vs. 5	0.206	.650

Note. * indicates the BIC-selected model within each group. BIC was the primary model-selection criterion; AIC and nested likelihood-ratio tests were supplementary. LL = log-likelihood; L. Ratio = likelihood ratio.

**Table 6:** Parameter estimates for the BIC-selected models of the attentional components.

Attention Network	Group	Term	Value	*SE*	*95% CI*	*t*	*p*
Executive control	Deaf	Intercept	0.203	0.006	[0.192, 0.215]	34.131	<.001
		Age	−0.005	0.002	[−0.009, −0.001]	−2.538	.012
	Hearing	Intercept	0.428	0.035	[0.360, 0.497]	12.274	<.001
		Log age	−0.118	0.014	[−0.145, −0.090]	−8.429	<.001
Orienting	Deaf	Intercept	0.012	0.005	[0.002, 0.021]	2.328	.021
		Age	0.007	0.002	[0.004, 0.010]	4.190	<.001
	Hearing	Intercept	0.051	0.005	[0.041, 0.060]	10.439	<.001
		Age	0.007	0.001	[0.005, 0.009]	7.852	<.001
		Age^2^	−0.001	0.0001	[−0.002, −0.000]	−3.741	<.001
Alerting	Deaf	Intercept	0.034	0.006	[0.021, 0.047]	5.192	<.001
	Hearing	Intercept	0.037	0.003	[0.032, 0.042]	13.73	<.001

*Note. SE = standard error; CI = confidence interval.*

## Data Availability

The raw data supporting the conclusions of this article will be made available by the authors on request.

## Supplementary Materials

The following supporting information can be downloaded at: https://www.mdpi.com/article/10.3390/jintelligence14100242/s1, Table S1: EZ-diffusion cell retention and trial counts; Table S2: Condition × Group interactions for EZ-diffusion parameters; Table S3: Continuous Group × Age sensitivity analyses; Table S4: Combined Group × Age models (GLS, ML): fixed-effect estimates; Table S5: Likelihood ratio tests: additive vs. interactive combined models; Table S6: Sex- and Raven-IQ-adjusted Group × Age Band interactions.

## Abbreviations

ACC	Anterior Cingulate Cortex
AIC	Akaike Information Criterion
ANT	Attention Network Test
BIC	Bayesian Information Criterion
CNV	Contingent Negative Variation
CSL	Chinese Sign Language
GLS	Generalized Least Squares
LRT	Likelihood Ratio Test
PFC	Prefrontal Cortex
