# Supplementary material for: Attention Networks in Deaf Youth: A Cross-Sectional Study of Age-Related Patterns
Source: J Intell. 2026 Oct 3;14(10):242. doi: 10.3390/jintelligence14100242 (PMC13706500; doi:10.3390/jintelligence14100242)
Supplement: Supplementary file 1 [file jintelligence-14-00242-s001.zip › jintelligence-4527302-supplementary.pdf]

Table S1. EZ-diffusion cell retention and trial counts

Network	Condition	Group	Expected	Observed	Missing	EZ retained	Median trials	Minimum correct
Orienting	center	Hearing	325	325	0	325	20	10
Executive control	congruent	Hearing	325	325	0	325	40	25
Alerting	double	Hearing	325	325	0	325	20	9
Executive control	incongruent	Hearing	325	325	0	325	40	12
Alerting	none	Hearing	325	325	0	325	20	10
Orienting	spatial	Hearing	325	325	0	325	20	11
Orienting	center	Deaf	200	200	0	200	20	10
Executive control	congruent	Deaf	200	200	0	200	40	30
Alerting	double	Deaf	200	200	0	200	20	10
Executive control	incongruent	Deaf	200	200	0	200	39	9
Alerting	none	Deaf	200	200	0	200	20	9
Orienting	spatial	Deaf	200	200	0	200	20	9

Note. All observed cells produced finite EZ parameters. Accuracy boundary corrections followed Wagenmakers et al. (2007).

Table S2. Condition × Group interactions for EZ-diffusion parameters

Network	Parameter	Covariate	*N*	*F*	*p*	p_{Holm}
Executive control	v	None	525	94.97	< .001	< .001
Executive control	v	Sex	525	94.86	< .001	< .001
Executive control	v	Raven IQ	525	94.83	< .001	< .001
Executive control	a	None	525	5.77	.017	.116
Executive control	a	Sex	525	5.76	.017	.117
Executive control	a	Raven IQ	525	5.78	.017	.116
Executive control	Ter	None	525	18.07	< .001	< .001
Executive control	Ter	Sex	525	18.05	< .001	< .001
Executive control	Ter	Raven IQ	525	18.09	< .001	< .001
Orienting	v	None	525	0.26	.613	.786
Orienting	v	Sex	525	0.26	.614	.786
Orienting	v	Raven IQ	525	0.26	.614	.786
Orienting	a	None	525	0.73	.393	.786
Orienting	a	Sex	525	0.73	.393	.786
Orienting	a	Raven IQ	525	0.73	.393	.786
Orienting	Ter	None	525	3.84	.051	.303
Orienting	Ter	Sex	525	3.85	.050	.301
Orienting	Ter	Raven IQ	525	3.83	.051	.304
Alerting	v	None	525	1.90	.168	.505
Alerting	v	Sex	525	1.90	.169	.506
Alerting	v	Raven IQ	525	1.90	.168	.505
Alerting	a	None	525	3.15	.077	.384
Alerting	a	Sex	525	3.14	.077	.385
Alerting	a	Raven IQ	525	3.14	.077	.385
Alerting	Ter	None	525	2.50	.114	.457
Alerting	Ter	Sex	525	2.50	.115	.458
Alerting	Ter	Raven IQ	525	2.50	.114	.457

Note. The Covariate column indicates whether the model was unadjusted or adjusted for sex or Raven IQ. The *p* column contains the uncorrected Condition × Group test, whereas p_{Holm} is adjusted across the nine diffusion-parameter interaction tests within each covariate specification.

Table S3. Continuous Group × Age sensitivity analyses

Outcome	Score	Covariate	N	L.Ratio (*2 df*)	*p*	p_{Holm}
executive control	Normalized RT contrast	None	525	4.98	.083	.166
executive control	Normalized RT contrast	Sex	525	4.77	.092	.184
executive control	Normalized RT contrast	Raven IQ	525	5.19	.075	.150
orienting	Normalized RT contrast	None	525	14.31	< .001	.002
orienting	Normalized RT contrast	Sex	525	14.54	< .001	.002
orienting	Normalized RT contrast	Raven IQ	525	14.33	< .001	.002
alerting	Normalized RT contrast	None	525	0.17	.917	.917
alerting	Normalized RT contrast	Sex	525	0.13	.939	.939
alerting	Normalized RT contrast	Raven IQ	525	0.19	.910	.910
raw_executive control	Raw RT difference (ms)	None	525	6.47	.039	.118
raw_executive control	Raw RT difference (ms)	Sex	525	6.41	.041	.122
raw_executive control	Raw RT difference (ms)	Raven IQ	525	6.62	.037	.111
raw_orienting	Raw RT difference (ms)	None	525	1.09	.581	1.000
raw_orienting	Raw RT difference (ms)	Sex	525	0.99	.611	1.000
raw_orienting	Raw RT difference (ms)	Raven IQ	525	1.10	.577	1.000
raw_alerting	Raw RT difference (ms)	None	525	0.85	.654	1.000
raw_alerting	Raw RT difference (ms)	Sex	525	0.83	.660	1.000
raw_alerting	Raw RT difference (ms)	Raven IQ	525	0.91	.634	1.000

Note. The Covariate column indicates model adjustment for sex or Raven IQ. The *p* column contains the uncorrected Group × Age test, whereas p_{Holm} is adjusted across the three attention networks separately for each scoring method and covariate specification.

Table S4. Combined Group × Age models (GLS, ML): fixed-effect estimates

Network	Model	Term	*b*	*SE*	*t*	*p*
Executive control	Additive	Intercept (deaf, at mean age)	0.2007	0.0061	32.889	< 0.001
Executive control	Additive	Hearing - Deaf (at mean age)	-0.0725	0.0071	-10.209	< 0.001
Executive control	Additive	Age (linear, deaf)	-0.0083	0.001	-8.1	< 0.001
Executive control	Additive	Age squared (deaf)	0.0005	0.0003	1.469	0.1425
Executive control	Interactive	Intercept (deaf, at mean age)	0.2054	0.0073	28.289	< 0.001
Executive control	Interactive	Hearing - Deaf (at mean age)	-0.0801	0.0097	-8.296	< 0.001
Executive control	Interactive	Age (linear, deaf)	-0.005	0.0019	-2.554	0.0109
Executive control	Interactive	Age squared (deaf)	-0.0003	0.0006	-0.459	0.6467
Executive control	Interactive	Hearing x Age	-0.0045	0.0023	-1.952	0.0515
Executive control	Interactive	Hearing x Age squared	0.0009	0.0007	1.355	0.1761
Executive control	Additive + Raven IQ	Intercept (deaf, at mean age)	0.2395	0.0303	7.913	< 0.001
Executive control	Additive + Raven IQ	Hearing - Deaf (at mean age)	-0.0705	0.0072	-9.742	< 0.001
Executive control	Additive + Raven IQ	Age (linear, deaf)	-0.0083	0.001	-8.079	< 0.001
Executive control	Additive + Raven IQ	Age squared (deaf)	0.0005	0.0003	1.584	0.1139
Executive control	Additive + Raven IQ	Raven IQ	-0.0008	0.0006	-1.309	0.1911
Executive control	Interactive + Raven IQ	Intercept (deaf, at mean age)	0.2471	0.031	7.974	< 0.001
Executive control	Interactive + Raven IQ	Hearing - Deaf (at mean age)	-0.079	0.0097	-8.161	< 0.001
Executive control	Interactive + Raven IQ	Age (linear, deaf)	-0.005	0.0019	-2.562	0.0107
Executive control	Interactive + Raven IQ	Age squared (deaf)	-0.0003	0.0006	-0.498	0.6189
Executive control	Interactive + Raven IQ	Hearing x Age	-0.0044	0.0023	-1.919	0.0556
Executive control	Interactive + Raven IQ	Hearing x Age squared	0.001	0.0007	1.484	0.1385
Executive control	Interactive + Raven IQ	Raven IQ	-0.0008	0.0006	-1.381	0.1678
Orienting	Additive	Intercept (deaf, at mean age)	0.0152	0.005	3.026	0.0026
Orienting	Additive	Hearing - Deaf (at mean age)	0.0275	0.0058	4.7	< 0.001
Orienting	Additive	Age (linear, deaf)	0.0074	0.0008	8.784	< 0.001
Orienting	Additive	Age squared (deaf)	-0.0004	0.0003	-1.684	0.0927
Orienting	Interactive	Intercept (deaf, at mean age)	0.003	0.0059	0.503	0.6151
Orienting	Interactive	Hearing - Deaf (at mean age)	0.0477	0.0079	6.051	< 0.001
Orienting	Interactive	Age (linear, deaf)	0.0063	0.0016	3.976	0.0001
Orienting	Interactive	Age squared (deaf)	0.0011	0.0005	2.233	0.026
Orienting	Interactive	Hearing x Age	0.001	0.0019	0.533	0.5943
Orienting	Interactive	Hearing x Age squared	-0.0021	0.0006	-3.787	0.0002
Orienting	Additive + Raven IQ	Intercept (deaf, at mean age)	0.0191	0.025	0.763	0.4458
Orienting	Additive + Raven IQ	Hearing - Deaf (at mean age)	0.0277	0.006	4.631	< 0.001
Orienting	Additive + Raven IQ	Age (linear, deaf)	0.0074	0.0008	8.778	< 0.001
Orienting	Additive + Raven IQ	Age squared (deaf)	-0.0004	0.0003	-1.661	0.0972
Orienting	Additive + Raven IQ	Raven IQ	-0.0001	0.0005	-0.157	0.8752
Orienting	Interactive + Raven IQ	Intercept (deaf, at mean age)	-0.0022	0.0253	-0.088	0.9297
Orienting	Interactive + Raven IQ	Hearing - Deaf (at mean age)	0.0476	0.0079	6.008	< 0.001
Orienting	Interactive + Raven IQ	Age (linear, deaf)	0.0063	0.0016	3.973	0.0001
Orienting	Interactive + Raven IQ	Age squared (deaf)	0.0011	0.0005	2.236	0.0258
Orienting	Interactive + Raven IQ	Hearing x Age	0.001	0.0019	0.527	0.5984
Orienting	Interactive + Raven IQ	Hearing x Age squared	-0.0022	0.0006	-3.786	0.0002
Orienting	Interactive + Raven IQ	Raven IQ	0.0001	0.0005	0.212	0.8323
Alerting	Additive	Intercept (deaf, at mean age)	0.0389	0.0054	7.208	< 0.001
Alerting	Additive	Hearing - Deaf (at mean age)	0.0057	0.0063	0.909	0.3638
Alerting	Additive	Age (linear, deaf)	0.0002	0.0009	0.265	0.7913
Alerting	Additive	Age squared (deaf)	-0.0006	0.0003	-2.165	0.0308
Alerting	Interactive	Intercept (deaf, at mean age)	0.0383	0.0064	5.943	< 0.001
Alerting	Interactive	Hearing - Deaf (at mean age)	0.0066	0.0086	0.767	0.4434
Alerting	Interactive	Age (linear, deaf)	-0.0004	0.0017	-0.203	0.8393
Alerting	Interactive	Age squared (deaf)	-0.0005	0.0005	-0.992	0.3217
Alerting	Interactive	Hearing x Age	0.0008	0.002	0.393	0.6942
Alerting	Interactive	Hearing x Age squared	-0.0001	0.0006	-0.189	0.8499
Alerting	Additive + Raven IQ	Intercept (deaf, at mean age)	0.0157	0.0268	0.588	0.5569
Alerting	Additive + Raven IQ	Hearing - Deaf (at mean age)	0.0046	0.0064	0.712	0.4766
Alerting	Additive + Raven IQ	Age (linear, deaf)	0.0002	0.0009	0.248	0.8041
Alerting	Additive + Raven IQ	Age squared (deaf)	-0.0006	0.0003	-2.237	0.0257
Alerting	Additive + Raven IQ	Raven IQ	0.0005	0.0005	0.882	0.3781
Alerting	Interactive + Raven IQ	Intercept (deaf, at mean age)	0.0145	0.0275	0.527	0.5983
Alerting	Interactive + Raven IQ	Hearing - Deaf (at mean age)	0.0059	0.0086	0.691	0.4899
Alerting	Interactive + Raven IQ	Age (linear, deaf)	-0.0003	0.0017	-0.199	0.8422
Alerting	Interactive + Raven IQ	Age squared (deaf)	-0.0005	0.0005	-0.966	0.3344
Alerting	Interactive + Raven IQ	Hearing x Age	0.0008	0.002	0.371	0.7107
Alerting	Interactive + Raven IQ	Hearing x Age squared	-0.0002	0.0006	-0.275	0.7836
Alerting	Interactive + Raven IQ	Raven IQ	0.0005	0.0005	0.889	0.3744

Note. Age is mean-centered; the deaf group is the reference level; n = 525 for each network.

Table S5. Likelihood ratio tests: additive vs. interactive combined models

**Network**	**N**	**Additive logLik (*df*)**	**Interactive logLik (*df*)**	***L.Ratio***	***df***	***p***	***p_{Holm}***
Executive control	525	600.963 (5)	603.453 (7)	4.981	2	0.0829	0.166
Orienting	525	702.769 (5)	709.926 (7)	14.314	2	0.000779	0.002
Alerting	525	666.111 (5)	666.198 (7)	0.174	2	0.917	0.917

Note. The p column contains the uncorrected Group × Age test, whereas p_{Holm} is adjusted across the three attention networks.

Table S6. Sex- and Raven-IQ-adjusted Group × Age Band interactions

Outcome	Covariate	N	*F(df1, df2)*	*p*	p_{Holm}
Executive control	Sex	525	2.94 (3, 516)	.033	.066
Executive control	Raven IQ	525	3.10 (3, 516)	.026	.053
orienting	Sex	525	10.90 (3, 516)	< .001	< .001
orienting	Raven IQ	525	10.81 (3, 516)	< .001	< .001
alerting	Sex	525	0.06 (3, 516)	.981	.981
alerting	Raven IQ	525	0.07 (3, 516)	.974	.974

Note. All models were adjusted for the covariate shown. The p column contains the uncorrected Group × Age Band interaction test, whereas p_{Holm} is adjusted across the three attention networks separately for each covariate.
